# How does burnout affect physician productivity? A systematic literature review

**DOI:** 10.1186/1472-6963-14-325

**Published:** 2014-07-28

**Authors:** Carolyn S Dewa, Desmond Loong, Sarah Bonato, Nguyen Xuan Thanh, Philip Jacobs

**Affiliations:** 1 Centre for Research on Employment and Workplace Health, Centre for Addiction and Mental Health, 33 Russell Street, Toronto, Ontario M5S 2S1, Canada; 2 Library Services, Centre for Addiction and Mental Health, 33 Russell Street, Toronto M5S 2S1, Canada; 3 Department of Psychiatry, University of Toronto, 250 College Street, Toronto M5T 1R8, Canada; 4 Institute of Health Economics, Edmonton T5J 3N4, Canada; 5 Department of Medicine, University of Alberta, Edmonton T6G 2R3, Canada

**Keywords:** Physician, Burnout, Productivity

## Abstract

**Background:**

Interest in the well-being of physicians has increased because of their contributions to the healthcare system quality. There is growing recognition that physicians are exposed to workplace factors that increase the risk of work stress. Long-term exposure to high work stress can result in burnout. Reports from around the world suggest that about one-third to one-half of physicians experience burnout. Understanding the outcomes associated with burnout is critical to understanding its affects on the healthcare system. Productivity outcomes are among those that could have the most immediate effects on the healthcare system. This systematic literature review is one of the first to explore the evidence for the types of physician productivity outcomes associated with physician burnout. It answers the question, “How does burnout affect physician productivity?”

**Methods:**

A systematic search was performed of: *Medline Current*, *Medline in process*, *PsycInfo*, *Embase* and *Web of Science*. The search period covered 2002 to 2012. The searches identified articles about practicing physicians working in civilian settings. Articles that primarily looked only at residents or medical students were excluded. Productivity was captured by hours worked, patients seen, sick leave, leaving the profession, retirement, workload and presenteeism. Studies also were excluded if: (1) the study sample was not comprised of at least 50% physicians, (2) the study did not examine the relationship between burnout and productivity or (3) a validated measure of burnout was not used.

**Results:**

The search identified 870 unique citations; 5 met the inclusion/exclusion criteria. This review indicates that globally there is recognition of the potential impact of physician burnout on productivity. Productivity was examined using: number of sick leave days, work ability, intent to either continue practicing or change jobs. The majority of the studies indicate there is a negative relationship between burnout and productivity. However, there is variation depending on the type of productivity outcome examined.

**Conclusions:**

There is evidence that burnout is associated with decreased productivity. However, this line of inquiry is still developing. A number of gaps are yet to be filled including understanding how to quantify the changes in productivity related to burnout.

## Background

There has been increasing interest in the well-being of physicians and their contributions to the quality of the healthcare system [1]. Part of this focus has been attributed to the recognition that physicians are exposed to workplace factors that increase the risk of work stress. Examples of these factors include long work hours [2], work overload [3], sleep deprivation and work conflicts [4]. In a meta-analysis, Alarcon [5] found that physician job demands, low job satisfaction and low organizational commitment are associated with emotional exhaustion among physicians. There is also recognition that long-term exposure to high work stress can result in burnout [6].

Reports from around the world suggest that about one-third to one-half of physicians of various specialties experience at least one dimension of burnout (e.g., [7-11]). Burnout has been conceptualized as a syndrome consisting of three dimensions: emotional exhaustion, depersonalization and low personal accomplishment [12]. Results of burnout include low job satisfaction [13,14], decreased mental health [15] and decreased quality of care [11].

As the literature examining the prevalence of burnout among physicians has increased, there also has been growing interest in understanding the outcomes associated with burnout. Identifying the outcomes associated with burnout is critical to understanding the scope of the problem as it affects the healthcare system. For example, Williams et al. [16] found that higher levels of perceived stress affect physician intention to withdraw from practicing. Other studies have observed burnout to be related to early retirement [17]. The losses in patient services related to work cutback and early retirement have been estimated to be at least CAN $213 million [18].

It has been asserted that there have been few studies examining the effectiveness of interventions designed to address burnout among physicians. A first step in filling this gap in the literature is to identify the types of outcomes that would be expected to change with the introduction of interventions. Of the types of outcomes that could be considered, productivity outcomes are among the outcomes that could have the most immediate effect on the healthcare system. This systematic literature review is one of the first to explore the evidence for the types of physician productivity outcomes associated with physician burnout. In it, we seek to answer the question, “How does burnout affect physician productivity?” In this review, we seek to identify how burnout affects the physician production of healthcare. This includes the ability to work, presence at work as well as workplace attachment.

## Methods

This systematic review was based on published peer-reviewed articles that were publically available. Thus, it was not submitted to an ethics board for review.

For the purposes of this systematic review, five databases were searched. They included: (1) *Medline Current* (contains journal citations and abstracts for biomedical literature in the fields of medicine, nursing, dentistry, veterinary medicine, the health care system, and the preclinical sciences), (2) *Medline in process* (contains journal citations and abstracts for biomedical literature that are in the process of being indexed in *Medline Current*), (3) *PsycINFO* (contains citations and summaries of journal articles, book chapters, books, technical reports, and dissertations, in the field of psychology and psychological aspects of related disciplines including medicine, psychiatry, nursing, sociology, education, pharmacology, physiology, linguistics, anthropology, sport, business, and law), (4) *Embase* (contains citations and abstracts of biomedical, drug-related and clinical literature), and (5) *Web of Science* (contains citations and abstracts from scholarly journals and conference proceedings in the sciences, social sciences, arts and humanities). The search period covered January 2002 to November 2012 and searches were limited to English language journals. The search was conducted between November 2012 and December 2012.

### Search strategy

The search strategy was developed and executed with the assistance of a professional health science librarian. (See Additional file 1 for the complete search strategy used in each database.) *Medline*, *Medline in Process*, *PsycINFO*, and *Embase* were searched using the OVID platform; while *Web of Science* was searched using the Thomson Reuters search interface. For the searches, burnout was defined as a syndrome of emotional exhaustion, cynicism (depersonalization) and reduced feelings of personal accomplishment related to work [12]. In addition, the searches focused on identifying articles about practicing physicians regardless of specialty who worked in civilian settings. As such, articles that primarily looked only at residents or medical students were excluded. However, search term limitations for residents and medical students were not used. This resulted in a broad search strategy to increase the likelihood that all studies on physician burnout would be found. Productivity was captured by hours worked, patients seen, sick leave, disability, quitting, leaving the profession, retirement, workload and presenteeism.

Review articles and commentaries were excluded, and the reference lists of relevant studies were hand searched.

### Screening process

Relevant articles were identified using a multi-phase screening process. To identify relevant articles, search results were screened first by title, then by abstract, and in the final screening process, by full-text review. Articles for which the title and abstract did not provide sufficient information to determine relevancy were considered in the full-text review. The multi-phase screening process was completed independently by two reviewers, CSD and DL. Inclusion criteria were: (1) the study examined the relationship between burnout and work productivity, (2) the sample population was comprised of practicing physicians regardless of specialty who worked in civilian settings. Exclusion criteria were: (1) the study sample was not comprised of at least 50% physicians, (2) the study did not examine the relationship between burnout and productivity or (3) a validated measure of burnout was not used (i.e., there was no evidence that the psychometric properties of the measure had been evaluated). The inter-rater reliability corrected for chance between CSD and DL was қ = 0.69. Articles for which there was initial disagreement were discussed until consensus was reached.

### Quality assessment

Articles that passed the three stage screening process moved on for quality assessment by the two reviewers (CSD and DL) using the following 10-item criteria adapted from Lagerveld et al. [19]:

(1) Study population is well described (e.g., age, sex, location of the study, physician specialty, practice location)

(2) Data collection methods are described

(3) Participation/response rate (at baseline) was at least 50%

(4) Burnout was assessed using a validated measure

(5) Productivity outcome was clearly defined

(6) Statistical method was appropriate for the question being answered

(7) Statistical significance of associations were tested and reported

(8) Study controlled for relevant confounding factors

(9) Number of cases in the analysis was at least 10 times the number of independent variables

(10) Research question was answered using longitudinal data (as opposed to cross-sectional data)

One point was awarded for each criterion that was met for a maximum score of 10. Studies that achieved a score between 1 and 4 were regarded as fair quality. Studies with scores between 5 and 8 were regarded as good quality, and studies that scored 9 or 10 were regarded as excellent.

## Results

### Description of inclusion and exclusion

**Figure 1:**
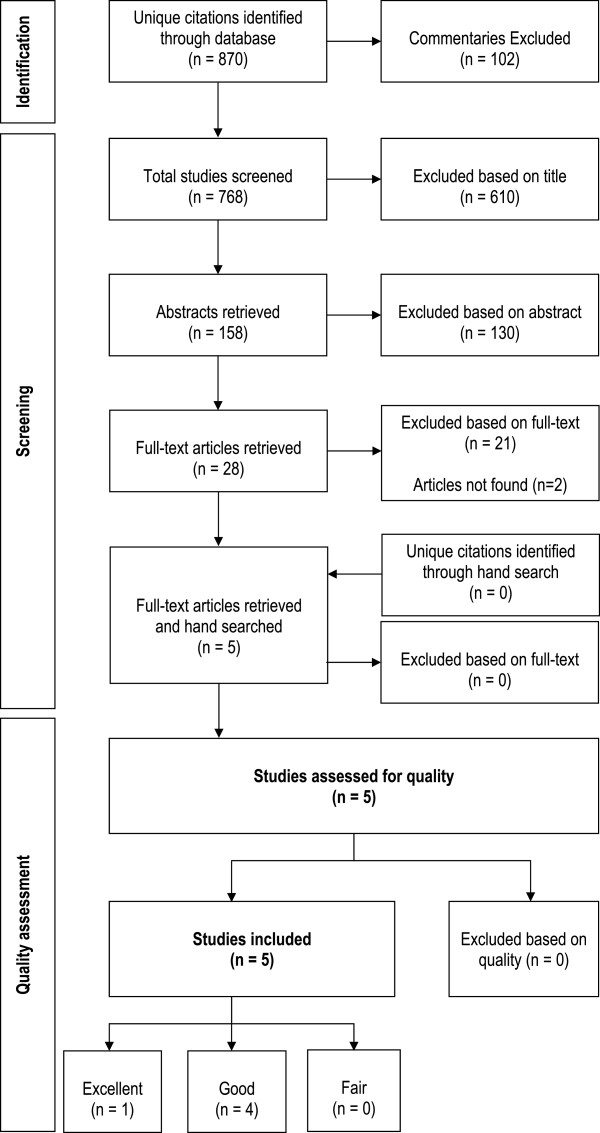
Flowchart of literature search results and inclusions/exclusions.

The electronic literature search resulted in the identification of 870 unique citations (Figure 1). Of these, 102 entries that were commentaries were excluded. Based on the title review, 610 citations were dropped. After the abstract review, another 130 citations were excluded; this left 28 articles for full-text review. After the full-text review, 5 articles remained and their reference lists were hand searched for relevant studies. No articles were identified in the hand search process. Reasons for the full-text review exclusions were: (1) the study sample was not comprised of at least 50% physicians (n = 2 articles), (2) did not examine the relationship between burnout and productivity (n = 21 articles) and (3) could not be obtained (n = 2 articles).

The five studies were conducted in the US, China, Hong Kong, The Netherlands as well as in 12 European countries: Bulgaria, Croatia, France, Greece, Hungary, Italy, Malta, Poland, Spain, Sweden, Turkey and the United Kingdom.

### Quality assessment

Based on the quality assessment, four of the five studies were categorized as good and one as excellent (Additional file 2, Additional file 3). The identified limitations were low response rates (<50%) (n = 3 studies), confounding factors not controlled for (n = 2 studies) and use of cross-sectional data (n = 5 studies).

### Overview of the studies

#### Burnout

Table 1 includes a summary of the studies. In 4 of the 5 studies, burnout was measured using the Maslach Burnout Inventory-HSS (MBI) [12]. The MBI measures three dimensions of burnout. These are Emotional Exhaustion (EE), Depersonalization (DP) and Personal Accomplishment (PA). One study used a measure developed by Pines et al. [20].

**Table 1:** Summary of articles

**Author(s)**	**Year**	**Location**	**Study population**	**Response rate**	**Sample size and characteristics**	**Burnout measure**	**Burnout Prevalence**
Hoff et al. [23]	2002	United States	Hospitalists (≥ 50% of time engaged in practice of general hospital medicine, or research and education related to general hospital medicine) who were members of the US National Association of Inpatient Physicians	48%	n = 393 hospitalists	21-item job burnout measure by Pines, Anderson, and Kafry (1981)	No risk of burnout = 59.1%
					≤5 years since graduation: over 25%		At risk of burnout = 27.6%
					Males: ~75%		Burned out = 13.4%
					Females: ~25%		
					Mean age = 40 yrs		
Ruitenburg et al. [21]	2012	The Netherlands	Hospital physicians working in one academic medical centre	51%	n = 422	Maslach Burnout Inventory	Medical doctor:
					Medical doctors: 54%		Mean EE = 13.3 ± 8.0
					Medical residents: 46%		Mean DP = 4.5 ± 4.1
					Medical doctors:		Burnout indicative = 6%
					Males: 52.0%		
					Females: 48.0%		
					Mean age = 47 ± 8.9 yrs		
					Years of practice = not reported		
Siu et al. [14]	2012	Hong Kong	1,000 public hospital doctors were randomly sampled from the 3,878 Hong Kong Public Doctors’ Association registry	23%	n = 226 physicians	Maslach Burnout Inventory	Mean Scores:
					Males: 66.8%		EE = 27.2 ± 13.2
					Females: 33.2%		DP = 10.9 ± 7.6
					Median age [Interquartile range] = 37.0 [30.5, 44.0] yrs		PA = 31.6 ± 8.8
					Median years of practice [Interquartile range] = 12.0 [6.0, 20.0]		
Soler et al. [22]	2008	12 European Countries: Bulgaria, Croatia, France, Greece, Hungary, Italy, Malta Poland, Spain, Sweden, Turkey, United Kingdom	Family Doctors who worked at least 50% of the time either in private practice or state employment. There is no information provided in the article regarding how the sample in each country was chosen.	41%	n = 1393 family doctors	Maslach Burnout Inventory	EE (95% CI):
					Males: 54.6%		
					Females: 45.4%		High = 43.0 (40.5, 45.6); Medium = 40.0 (37.5, 42.6); Low = 17.0 (15.1, 19.0)
					Mean age = 45.4 ± 8.5 yrs		DP (95% CI):
					Mean years since graduation = 19.2 ± 8.5		High = 35.3 (32.9, 37.9); Medium = 27.2 (24.9, 29.6); Low = 37.5 (35.0, 40.0)
							PA (95% CI):
							High = 32.0 (29.6, 34.5); Medium = 28.5 (26.2, 30.9); Low = 39.5 (37.0, 42.1)
Zhang & Feng [24]	2011	China	Randomly selected physicians practicing in one of 67 state-owned medical institutions in Hubei province. The sample included medical assistants, residents, attendings, associate chiefs and chiefs.	94%	n = 1451 physicians	Maslach Burnout Inventory	Not reported
					Males: 66.2%		
					Females: 33.8%		
					Age:		
					≤ 30 yrs = 38.3%		
					31-40 yrs = 37.2%		
					41-50 yrs = 16.6%		
					≥ 51 yrs = 7.9%		
					Years of service:		
					≤ 5 yrs = 37.9%		
					6-15 yrs = 41.6%		
					16-25 yrs = 16.0%		
					≥ 26 yrs = 4.5%		

Note: EE = Emotional Exhaustion; DP = Depersonalization; PA = Personal Achievement.

The rates of burnout in the study samples varied. Ruitenburg et al. [21] observed a burnout rate of 6% in their sample of Dutch hospital physicians. In contrast, Soler and colleagues [22] observed that 43% of their sample of family doctors from 12 European countries had high EE, 35% had high DP and 39% had low PA. Siu et al. [14] observed that 31% of their Hong Kong sample of public hospital doctors experienced burnout. In their sample of US hospitalists, Hoff et al. [23] reported that 13% of respondents had burnout.

#### Productivity measures

The productivity outcomes that were reported in the articles included number of sick leave days, intent to continue practicing, intent to change jobs, and work ability (Table 2). All of the studies reported significant negative relationships between the three dimensions of burnout and the productivity measures used.

**Table 2:** Productivity outcomes

**Author(s)**	**Number of sick leave days**	**Intent to continue practicing**	**Intent to change jobs**	**Work ability**
Hoff et al. [23]		**Years Intending to Remain a Hospitalist by Risk of Burnout**		
		No Risk of Burnout (n = 225)		
		< 4 years = 6.4%		
		4–10 years = 34.9%		
		> 10 years = 58.7%		
		At risk of burnout (n = 105)		
		< 4 years = 16.5%		
		4–10 years = 47.6%		
		> 10 years = 35.9%		
		Burned out (n = 51)		
		< 4 years = 44.0%		
		4–10 years = 24.0%		
		> 10 years = 22.0%		
Ruitenburg et al. [21]				Insufficient Workability with High Burnout:
				Odds Ratio (95% CI) = 9.5 (3.0, 30.6)
				p<0.001
Siu et al. [14]	**Median Sick Leave Days in the Last Year by Burnout [Interquartile range]**			
	High burnout = 1 [0, 3.0]			
	Non-high burnout = 0.25 [0, 2.0]			
	p = 0.127			
Soler et al. [22]	**Sick Leave in the Last Year by Burnout Dimensions**		**Seriously Considered Changing Jobs at Least Once over Past Months by Burnout Dimensions**	
	High EE (95% CI):		High EE (95% CI):	
	0 days = 37.9% (33.7, 42.3)		Thoughts of changing job = 66.4% (60.5, 71.8)	
	1-2 days = 52.3% (41.2, 63.2)		p < 0.001	
	≥ 3 days = 50.2% (42.6, 57.7)		High DP:	
	p < 0.001		Thoughts of changing job = 47.1% (41.1, 53.2)	
	High DP (95% CI):		p < 0.001	
	0 days = 31.3% (27.4, 35.6)		Low PA (95% CI):	
	1-2 days = 41.5% (31.1, 52.8)		Thoughts of changing jobs = 42.3% (36.5, 48.4)	
	≥ 3 days = 39.9% (32.7, 47.5)		p < 0.001	
	p < 0.01			
	Low PA (95% CI):			
	0 days = 29.9% (26.0, 34.1)			
	1-2 days = 28.5% (19.5, 39.5)			
	≥ 3 days = 38.9% (31.8, 46.6)			
	p < 0.05			
Zhang & Feng [24]			**Association between Burnout and Intent to Change Jobs**	
			EE Correlation: 0.229	
			p < 0.001	
			DP Correlation: 0.211	
			p < 0.001	
			Reduced PA Correlation: 0.114	
			p < 0.001	

Note: EE = Emotional Exhaustion; DP = Depersonalization; PA = Personal Achievement.

##### Sick leave

There are conflicting results reported in the literature regarding the use of sick leave days. One study based on responses from family doctors from 12 European countries found that all three burnout dimensions were significantly associated with more use of sick leave days [22]. Soler and colleagues [22] found that those who had at least one sick leave day during the year had significantly higher odds of reporting either high EE, DP or low PA. In contrast, Siu et al. [14] reported no statistically significant differences in the average sick leave days in the past year for public hospital physicians with and without high burnout scores.

##### Intent to change jobs

Studies also reported that high burnout scores are significantly associated with intentions to leave a current position. For example, Soler et al.’s [22] 12 European country study also found that those who affirmatively answered the question, “Have you seriously considered changing your job at least once over the past months?” had significantly greater odds of reporting either high EE, DP or low PA.

In their study, Zhang and colleagues [24] also found a significant relationship between burnout and intention of looking for another job. Their survey of physicians practicing in one of three hospitals in Hubei province in China indicated there are significant correlations between high EE, DP and low PA and the intention of looking for another job.

##### Intent to continue to practice medicine

There is also indication that burnout is significantly associated with intentions of quitting the current specialty. This could be problematic in systems in which there is a shortage of physicians or particular specialities. A survey of US hospitalists found that over 50% of respondents who were not at risk of burnout planned on continuing with hospital-based practice for more than 10 years [23]. In contrast, a little more than a third of hospitalists who were at risk of burnout reported an intention of continuing with hospital-based practice for more than 10 years. Among those who met criteria for burnout, about 44% indicated they intended to continue to practice as a hospitalist for less than 4 years.

##### Work ability

A study from one academic medical centre in the Netherlands found that hospital-based physicians who had high scores for both the EE and the DP dimensions of burnout had significantly greater odds of having self-perceived “insufficient” work ability [21]. Ruitenberg and colleagues [21] defined work ability as the “degree to which a worker is physically and mentally able to cope with the demands at work” (p. 2).

## Discussion

This review indicates that countries all over the world are beginning to recognize the potential impact of physician burnout on productivity. In the studies that were identified, productivity was examined in four different ways that included number of sick leave days, intent to continue practicing, intent to change jobs, and work ability. The results of the studies indicate that there is a negative relationship between burnout and productivity. However, there was at least one discrepancy depending on the type of productivity outcome of interest. For example, Soler et al. [22] found a significant relationship between burnout and sick leave while Siu et al. [14] did not. This may be because the type of productivity decrease chosen by physicians who experience burnout may be related to the context in which they practice. That is, one system may offer the option of taking sick leave while in another it may be more discouraged. This suggests that rather than one measure of productivity, it is important to consider several depending on the context in which the study is being conducted. At the same time, to help facilitate the translation of results to other systems, it may be important to consider a variety of productivity outcomes in the evaluation of physician burnout interventions.

Research regarding the relationship between burnout and productivity loss seems to be in its infancy. There are a number of questions that remain to be answered. For example, to what extent are the results generalizable to all physicians? Four of the reviewed studies focused on physicians primarily practicing in hospital-based settings. In addition, these studies did not report whether there were differences in productivity outcomes by specialty within the hospital setting. If there are differences in job characteristics by specialty that affect burnout, reduction in productivity may not be entirely attributable to burnout but to the type of specialty as well.

Furthermore, two of the studies in this review included residents in their samples. This may have affected results. Because they are still in training, residents may have different experiences that may in turn affect the relationship between productivity and burnout. For example, if burnout is related to prolonged exposure to particular work factors, residents will have less exposure. Or, they may react differently from someone who has been practicing for a longer period of time.

Another challenge of applying this literature is related to the differences in the healthcare systems. For example, are there characteristics of China’s state-run system that may not exist in non-state run settings? To improve the generalizability of future studies, it would be useful if they included more information about the healthcare system context in which the physicians practice.

An additional limitation of the studies relates to the questions that ask about intent to either leave the current position or the specialty. These types of questions only gather information about intent rather than action. To the extent that intent develops into action, the measure is accurate. Otherwise, the measure could overestimate the number of people who actually follow through with their intentions.

Although work ability is a promising measure of productivity, it is difficult to quantify the actual impact on productivity. Is it possible for someone who has a low work ability to be as productive as someone who has a high ability? The question remains to what extent does work ability correlate with productivity.

All of the studies used cross-sectional data. Consequently, it is difficult to assign causality. For example, Soler et al. [22] examined the relationship between dimensions of burnout and the productivity outcomes by using the burnout dimensions as the dependent variables in regression analyses. This could also indicate that the experience of burnout is related to productivity rather than vice versa.

Finally, there may be a publication bias. There may be studies which have not found significant relationships between burnout and productivity outcomes that may not have been published. If this is the case for a large number of studies, the bias could lead to an exaggeration of the relationship between burnout and productivity.

It should also be noted that there may be limitations to our search. While five databases were searched, it is possible that an article could have been missed if it did not appear in any of the databases. However, that possibility is small given the broad scope of each of the databases. Another limitation is that the search does not include research that was not published in English. However, it should be noted that despite the language constraint, the included studies came from both Europe and Asia. This suggests that at least some of the researchers from countries in which English is not a first language are publishing in English language journals.

## Conclusions

The findings of this systematic literature review indicate that there is interest throughout the world about the potential impact of physician burnout on productivity. There is evidence that burnout is associated with decreased productivity as it is captured in intent to leave a position or the field.

However, this line of inquiry is still developing. There are a number of gaps that are yet to be filled. These include understanding whether there are differences by specialty, how the healthcare system impacts burnout and productivity, and how to quantify the changes in productivity related to burnout.

## Abbreviations

EE: Emotional exhaustion; DP: Depersonalization; PA: Personal accomplishment.

## Competing interests

The authors declare that they have no competing interests.

## Authors’ contributions

CSD led the conception, design, data acquisition, analysis and interpretation of the data. DL collaborated on the design, data acquisition and analysis. SB collaborated on the design and data acquisition. NXT collaborated on the interpretation of the data. PJ collaborated on the conception and interpretation of data. All authors read and approved the final manuscript.

## Pre-publication history

The pre-publication history for this paper can be accessed here:

http://www.biomedcentral.com/1472-6963/14/325/prepub

## Supplementary Material

Additional file 1 Search terms used in search strategy. Click here for file

Additional file 2 **Quality assessment checklist.** The additional file contains the quality checklist criteria used to determine the quality of papers being analyzed for the systematic literature review and the scores for each article. Click here for file

Additional file 3 PRISMA 2009 Checklist. Click here for file

## References

[B1] Wallace JE Lemaire J Physician well being and quality of patient care: an exploratory study of the missing link Psychol Health Med 2009 14 5 545 552 10.1080/13548500903012871 19844833

[B2] Martin S More hours, more tired, more to do: results from the CMA’s 2002 Physician Resource Questionnaire Can Med Assoc J 2002 14 5 521 522 12240823 PMC121979

[B3] Virtanen P Oksanen T Kivimaki M Virtanen M Pentti J Vahtera J Work stress and health in primary health care physicians and hospital physicians Occup Environ Med 2008 14 5 364 366 10.1136/oem.2007.034793 18045846

[B4] Richter A Kostova P Baur X Wegner R Less work: more burnout? A comparison of working conditions and the risk of burnout by German physicians before and after the implementation of the EU Working Time Directive Int Arch Occup Environ Health 2014 14 2 205 215 10.1007/s00420-013-0849-x 23423279

[B5] Alarcon GM A meta-analysis of burnout with job demands, resources, and attitudes J Vocat Behav 2011 14 2011 549 562

[B6] Maslach C Leiter MP The truth about burnout 1997 San Francisco, CA: Jossey-Bass

[B7] Allegra CJ Hall R Yothers G Prevalence of burnout in the U.S. Oncology community: results of a 2003 survey J Oncol Pharm Pract/Am Soc Clin Oncol 2005 14 4 140 147 10.1200/JOP.1.4.140 PMC2794568 20871697

[B8] Arigoni F Bovier PA Sappino AP Trend of burnout among Swiss doctors Swiss Med Wkly 2010 14 w13070 20809437 10.4414/smw.2010.13070

[B9] Elit L Trim K Mand-Bains IH Sussman J Grunfeld E Society of Gynecologic Oncology C Job satisfaction, stress, and burnout among Canadian gynecologic oncologists Gynecol Oncol 2004 14 1 134 139 10.1016/j.ygyno.2004.04.014 15262131

[B10] Embriaco N Azoulay E Barrau K Kentish N Pochard F Loundou A Papazian L High level of burnout in intensivists: prevalence and associated factors.[Erratum appears in Am J Respir Crit Care Med. 2007 Jun 1;175(11):1209–10] Am J Respir Crit Care Med 2007 14 7 686 692 10.1164/rccm.200608-1184OC 17234905

[B11] Klein J Grosse Frie K Blum K von dem Knesebeck O Burnout and perceived quality of care among German clinicians in surgery International J Qual Health Care 2010 14 6 525 530 10.1093/intqhc/mzq056 20935011

[B12] Maslach C Jackson SE The measurement of experienced burnout J Occup Behav 1981 14 99 113 10.1002/job.4030020205

[B13] Sharma A Sharp DM Walker LG Monson JRT Stress and burnout in colorectal and vascular surgical consultants working in the UK National Health Service Psychooncology 2008 14 6 570 576 10.1002/pon.1269 17935146

[B14] Siu CFY Yuen SK Cheung A Burnout among public doctors in Hong Kong: cross-sectional survey Hong Kong Med J 2012 14 3 186 192 22665681

[B15] Asai M Morita T Akechi T Sugawara Y Fujimori M Akizuki N Nakano T Uchitomi Y Burnout and psychiatric morbidity among physicians engaged in end-of-life care for cancer patients: a cross-sectional nationwide survey in Japan Psychooncology 2007 14 5 421 428 10.1002/pon.1066 16929464

[B16] Williams ES Konrad TR Scheckler WE Pathman DE Linzer M McMurray JE Gerrity M Schwartz M Understanding physicians’ intentions to withdraw from practice: the role of job satisfaction, job stress, mental and physical health. 2001 Health Care Manag Rev 2010 14 2 105 115 10.1097/01.HMR.0000304509.58297.6f 20234217

[B17] Henkens K Leenders M Burnout and older workers’ intentions to retire Int J Manpow 2010 14 3 306 321 10.1108/01437721011050594

[B18] Dewa CS Jacobs P Thanh NX Loong D An estimate of the cost of burnout on early retirement and reduction in clinical hours of practicing physicians in Canada BMC Health Serv Res 2014 14 254 10.1186/1472-6963-14-254 24927847 PMC4062768

[B19] Lagerveld SE Bultmann U Franche RL van Dijk FJ Vlasveld MC van der Feltz-Cornelis CM Bruinvels DJ Huijs JJ Blonk RW van der Klink JJ Nieuwenhuijsen K Factors associated with work participation and work functioning in depressed workers: a systematic review J Occup Rehabil 2010 14 3 275 292 10.1007/s10926-009-9224-x 20091105 PMC2923705

[B20] Pines AM Aronson E Kafry D Burn Out: From Tedium to Personal Growth 1981 New York: Free Press

[B21] Ruitenburg MM Frings-Dresen MHW Sluiter JK The prevalence of common mental disorders among hospital physicians and their association with self-reported work ability: a cross-sectional study BMC Health Serv Res 2012 14 292 10.1186/1472-6963-12-292 22938170 PMC3459739

[B22] Soler JK Yaman H Esteva M Dobbs F Asenova RS Katic M Ozvacic Z Desgranges JP Moreau A Lionis C Kotanyi P Carelli F Nowak PR de Aguiar Sa Azeredo Z Marklund E Churchill D Ungan M European General Practice Research Network Burnout Study Group Burnout in European family doctors: the EGPRN study Fam Pract 2008 14 4 245 265 10.1093/fampra/cmn038 18622012

[B23] Hoff T Whitcomb WF Nelson JR Thriving and surviving in a new medical career: the case of hospitalist physicians J Health Soc Behav 2002 14 1 72 91 10.2307/3090246 11949198

[B24] Zhang Y Feng X The relationship between job satisfaction, burnout, and turnover intention among physicians from urban state-owned medical institutions in Hubei China: a cross-sectional study BMC Health Serv Res 2011 14 235 10.1186/1472-6963-11-235 21943042 PMC3197494

