# Supplementary material for: How does burnout affect physician productivity? A systematic literature review
Source: BMC Health Serv Res. 2014 Jul 28;14:325. doi: 10.1186/1472-6963-14-325 (PMC4119057; doi:10.1186/1472-6963-14-325)
Supplement: Additional file 1 — Search terms used in search strategy. [file 1472-6963-14-325-S1.pdf]

Search terms used in search strategy

Database	Search Terms
Medline Current	[exp Burnout, Professional/ OR burnout.mp. OR (burnout adj3 effect*).mp.] **AND** [exp Physicians/ OR exp Psychiatry/ OR allergist*.mp. OR anesthesiologist*.mp. OR cardiologist*.mp. OR clinical pharmacologist*.mp. OR clinical toxicologist*.mp. OR dermatologist*.mp. OR doctor*.mp. OR endocrinologist*.mp. OR gastroenterologist*.mp. OR gynecologist*.mp. OR hematologist*.mp. OR immunologist*.mp. OR medical biochemist*.mp. OR medical geneticist*.mp. OR medical microbiologist*.mp. OR nephrologist*.mp. OR neurologist*.mp. OR neuropathologist*.mp. OR neuroradiologist*.mp. OR occupational physician*.mp. OR oncologist*.mp. OR ophthalmologist*.mp. OR pathologist*.mp. OR pediatrician*.mp. OR physician*.mp. OR psychiatrist*.mp. OR radiologist*.mp. OR rheumatologist*.mp. OR surgeon*.mp. OR urologist*.mp.] **AND** [exp Professional Impairment/ OR exp Sick Leave/ OR exp Absenteeism/ OR exp Personnel Turnover/ OR exp Workload/ OR exp Patient Care Management/ OR (impair* adj3 physician*).mp. OR (impair* adj3 doctor*).mp. OR (sick* adj3 day*).mp. OR (sick* adj3 leave*).mp. OR (illness adj3 day*).mp. OR quit*.mp. OR (patient adj3 volume*).mp. OR (patient adj3 load*).mp. OR (patient* adj3 care).mp. OR (patient* adj3 caring).mp. OR presenteeism*.mp. OR turnover*.mp. OR workload*.mp.]
Medline In-process	[exp Burnout, Professional/ OR burnout.mp. OR (burnout adj3 effect*).mp.] **AND** [exp Physicians/ OR exp Psychiatry/ OR allergist*.mp. OR anesthesiologist*.mp. OR cardiologist*.mp. OR clinical pharmacologist*.mp. OR clinical toxicologist*.mp. OR dermatologist*.mp. OR doctor*.mp. OR endocrinologist*.mp. OR gastroenterologist*.mp. OR gynecologist*.mp. OR hematologist*.mp. OR immunologist*.mp. OR medical biochemist*.mp. OR medical geneticist*.mp. OR medical microbiologist*.mp. OR nephrologist*.mp. OR neurologist*.mp. OR neuropathologist*.mp. OR neuroradiologist*.mp. OR occupational physician*.mp. OR oncologist*.mp. OR ophthalmologist*.mp. OR pathologist*.mp. OR pediatrician*.mp. OR physician*.mp. OR psychiatrist*.mp. OR radiologist*.mp. OR rheumatologist*.mp. OR surgeon*.mp. OR urologist*.mp.] **AND** [exp Professional Impairment/ OR exp Sick Leave/ OR exp Absenteeism/ OR exp Personnel Turnover/ OR exp Workload/ OR exp Patient Care Management/ OR (impair* adj3 physician*).mp. OR (impair* adj3 doctor*).mp. OR (sick* adj3 day*).mp. OR (sick* adj3 leave*).mp. OR (illness adj3 day*).mp. OR quit*.mp. OR (patient adj3 volume*).mp. OR (patient adj3 load*).mp. OR (patient* adj3 care).mp. OR (patient* adj3 caring).mp. OR presenteeism*.mp. OR turnover*.mp. OR workload*.mp.]
PsycINFO	[burnout.mp. OR (burnout adj3 effect*).mp.] **AND** [exp physicians/ OR exp clinicians/ OR exp Psychiatry/ OR allergist*.mp. OR anesthesiologist*.mp. OR cardiologist*.mp. OR clinical pharmacologist*.mp. OR clinical toxicologist*.mp. OR dermatologist*.mp. OR doctor*.mp. OR endocrinologist*.mp. OR gastroenterologist*.mp. OR gynecologist*.mp. OR hematologist*.mp. OR immunologist*.mp. OR medical biochemist*.mp. OR medical geneticist*.mp. OR medical microbiologist*.mp. OR nephrologist*.mp. OR neurologist*.mp. OR neuropathologist*.mp. OR neuroradiologist*.mp. OR occupational physician*.mp. OR oncologist*.mp. OR ophthalmologist*.mp. OR pathologist*.mp. OR pediatrician*.mp. OR physician*.mp. OR psychiatrist*.mp. OR radiologist*.mp. OR rheumatologist*.mp. OR surgeon*.mp. OR urologist*.mp.] **AND** [exp impaired professionals/ OR exp employee leave benefits/ OR exp employee absenteeism/ OR absenteeism*.mp. OR exp employee turnover/ OR exp work load/ OR (patient* adj3 car* adj3 manag*).mp. OR (impair* adj3 physician*).mp. OR (impair* adj3 doctor*).mp. OR (sick* adj3 day*).mp. OR (sick* adj3 leave*).mp. OR (illness adj3 day*).mp. OR quit*.mp. OR (patient adj3 volume*).mp. OR (patient adj3 load*).mp. OR (patient* adj3 care).mp. OR (patient* adj3 caring).mp. OR presenteeism*.mp. OR turnover*.mp. OR workload*.mp.]

Database	Search Terms
Embase	[exp Burnout/ OR burnout.mp. OR (burnout adj3 effect*).mp.] **AND** [exp Physicians/ OR exp Psychiatry/ OR allergist*.mp. OR anesthesiologist*.mp. OR cardiologist*.mp. OR clinical pharmacologist*.mp. OR clinical toxicologist*.mp. OR dermatologist*.mp. OR doctor*.mp. OR endocrinologist*.mp. OR gastroenterologist*.mp. OR gynecologist*.mp. OR hematologist*.mp. OR immunologist*.mp. OR medical biochemist*.mp. OR medical geneticist*.mp. OR medical microbiologist*.mp. OR nephrologist*.mp. OR neurologist*.mp. OR neuropathologist*.mp. OR neuroradiologist*.mp. OR occupational physician*.mp. OR oncologist*.mp. OR ophthalmologist*.mp. OR pathologist*.mp. OR pediatrician*.mp. OR physician*.mp. OR psychiatrist*.mp. OR radiologist*.mp. OR rheumatologist*.mp. OR surgeon*.mp. OR urologist*.mp.] **AND** [exp medical leave/ OR exp Absenteeism/ OR exp turnover time/ OR exp Workload/ OR exp patient care/ OR (impair* adj3 physician*).mp. OR (impair* adj3 doctor*).mp. OR (sick* adj3 day*).mp. OR (sick* adj3 leave*).mp. OR (illness adj3 day*).mp. OR quit*.mp. OR (patient adj3 volume*).mp. OR (patient adj3 load*).mp. OR (patient* adj3 care).mp. OR (patient* adj3 caring).mp. OR presenteeism*.mp. OR turnover*.mp. OR workload*.mp.]
Web of Science	[burnout*] **AND** [physician* OR clinician* OR Psychiatry OR allergist* OR anesthesiologist* OR cardiologist* OR clinical pharmacologist* OR clinical toxicologist* OR dermatologist* OR doctor* OR endocrinologist* OR gastroenterologist* OR gynecologist* OR hematologist* OR immunologist* OR medical biochemist* OR medical geneticist* OR medical microbiologist* OR nephrologist* OR neurologist* OR neuropathologist* OR neuroradiologist* OR occupational physician* OR oncologist* OR ophthalmologist* OR pathologist* OR pediatrician* OR physician* OR psychiatrist* OR radiologist* OR rheumatologist* OR surgeon* OR urologist*] **AND** [professional impair* OR impair* prefessional* OR sick leav* OR exp employ* leave benefit* OR absen* OR turnover* OR workload* OR work load* OR patient* car* manag* OR impair* physician* OR impair* doctor* OR sick* day* OR sick* leave* OR illness day* OR quit* OR patient* volume* OR patient* load* OR patient* care* OR patient* caring OR presenteeism*]
