# Supplementary material for: How does burnout affect physician productivity? A systematic literature review
Source: BMC Health Serv Res. 2014 Jul 28;14:325. doi: 10.1186/1472-6963-14-325 (PMC4119057; doi:10.1186/1472-6963-14-325)
Supplement: Additional file 2 — Quality assessment checklist. The additional file contains the quality checklist criteria used to determine the quality of papers being analyzed for the systematic literature review and the scores for each article. [file 1472-6963-14-325-S2.pdf]

Quality Assessment Checklist

Author(s)	Year	1	2	3	4	5	6	7	8	9	10
Hoff et al. [23]	2002	1	1	0	1	1	1	1	1	1	0
Ruitenburg et al. [21]	2012	1	1	1	1	1	1	1	0	1	0
Siu et al. [14]	2012	1	1	0	1	1	1	1	0	1	0
Soler et al. [22]	2008	1	1	0	1	1	1	1	1	1	0
Zhang & Feng [24]	2011	1	1	1	1	1	1	1	1	1	0

Quality Assessment Criteria

1. Study population well described (e.g., age, sex, location of the study, physician specialty, practice location)
2. Data collection methods described
3. Participation/response rate (at baseline) at least 50%
4. Burnout is assessed using a validated measure
5. Productivity outcome clearly defined
6. Statistical method is appropriate for question being answered
7. Statistical significance of associations are tested and reported
8. Study controls for relevant confounding factors
9. Number of cases in the analysis is at least 10 times the number of independent variables
10. Research question is answered using longitudinal data (as opposed to cross-sectional data)
